# Probiotics Do Not Alter the Long-Term Stability of the Supragingival Microbiota in Healthy Subjects: A Randomized Controlled Trial

**DOI:** 10.3390/pathogens10040391

**Published:** 2021-03-24

**Authors:** Christine Lundtorp-Olsen, Christian Enevold, Svante Twetman, Daniel Belstrøm

**Affiliations:** 1 Department of Odontology, Section for Clinical Oral Microbiology, Faculty of Health and Medical Sciences, University of Copenhagen, 2200 Copenhagen, Denmark; christine-lundtorp@hotmail.com (C.L.-O.); stwe@sund.ku.dk (S.T.); 2 Center for Rheumatology and Spine Diseases, Rigshospitalet, Institute for Inflammation Research, Copenhagen University Hospital, 2100 Copenhagen, Denmark; chrej@yahoo.dk

**Keywords:** oral microbiota, 16S rDNA, homeostasis, probiotics

## Abstract

Background: The purpose of the present study was to longitudinally characterize the supragingival microbiota throughout a three months period in orally healthy individuals. We tested the hypothesis that the supragingival microbiota shows a high degree of compositional stability, which is resilient against the external perturbation of regular use of probiotics, as long as oral health is maintained. Methods: The present study was a double-blinded, randomized, placebo-controlled clinical trial. The study population comprised a total of 110 oral and systemic healthy individuals, distributed in a probiotic (*n* = 55) and placebo (*n* = 55) group, where the test group consumed tablets with the probiotic strains *Lacticaseibacillus* *rhamnosus* (formerly *Lactobacillus*) PB01 DSM14870 and *Latilactobacillus curvatus* (formerly *Lactobacillus*) EB10 DSM32307 for a period of 12 weeks. Supragingival plaque samples and clinical registrations were performed at baseline, and after 4, 8, and 12 weeks, respectively. The supragingival microbiota was characterized by means of 16S rDNA sequencing. Sequences were referenced against the HOMD database. Results: No significant changes of the core microbiota, as expressed by relative abundance of predominant genera and species were evident during the three months observation period in the probiotic or the placebo group. Conclusions: Data from the present study clearly demonstrate long term compositional stability of the supragingival microbiota as long as oral health is maintained. In addition, the tested probiotics had no augmenting effect on the supragingival microbiota in oral health.

## 1. Introduction

The oral cavity constitutes a complex open-ended ecosystem, where symbiotic interactions of the oral microbiota and the host’s immune system support oral health. Indeed, ecological perturbations which affect either structure or function of the oral microbiota can induce dysbiosis in the local environment, which may lead to development of oral diseases such as dental caries and periodontitis [1,2].

The oral microbiota is constituted by a diverse range of bacteria comprising more than 700 unique species [3], and the composition of the oral microbiota is a reflection of different ecological niches found in the oral cavity [4,5]. For example, the teeth are characterized by non-shedding enamel surfaces, which is why the teeth offer almost ideal conditions for the formation of a diverse supragingival biofilm [6]. Indeed, the supragingival biofilm constitutes a complex microcosmos, which to some extend express resistance and plasticity, towards transient ecological challenges [6].

Smoking [7,8], diet [9], and absence of adequate oral hygiene [10] are examples of ecological stress factors, which favor growth of parts of the microbiota on expense of others. Therefore, long term exposure to such perturbations is reflected by compositional changes in the oral microbiota. In line, presence of treatment-requiring oral diseases, as exemplified by dental caries and periodontitis, associates with compositional changes of the oral microbiota [11,12,13]. Notably, compositional changes of the oral microbiota seem to reflect the nature of the perturbation, with dental caries and periodontitis having almost antagonistic impacts on composition of the oral microbiota [14,15,16]. Therefore, compositional changes of the oral microbiota have been suggested as a potential biomarker, which could ultimately be used to identify biological trajectories associated with increased risk of developing oral diseases. However, a prerequisite for implementation of such an approach is to document that the oral microbiota expels long-term stability in oral health.

Probiotics have been suggested to support oral health, through a proposed direct effect on the microbial ecosystem, as well as a systemic indirect interaction with the immune system of the host [17]. During recent years, several epidemiological studies have investigated the impact of probiotics on oral diseases, i.e., caries, gingivitis, periodontitis, and oral candidiasis [18,19,20,21,22,23,24,25,26], and few studies have investigated the impact of probiotics on the oral microbiota [27,28,29,30]. However, to the best of our knowledge, it remains to be elucidated, if long term regular use of probiotics can actually induce compositional changes to the oral microbiota in orally and systemically healthy individuals.

The aim of the present study was therefore twofold. First, we wanted to test if the composition of the supragingival microbiota shows long-term stability. Secondary, the aim was to learn if daily use of probiotics *Lacticaseibacillus rhamnosus* (formerly *Lactobacillus*) PB01 DSM14870 and *Latilactobacillus curvatus* (formerly *Lactobacillus*) EB10 DSM32307 has any impact on the composition of the supragingival microbiota in healthy conditions. Accordingly, we conducted a randomized double-blinded clinical trial in orally and systemically healthy individuals, to test the hypothesis that the composition of the supragingival microbiota shows long-term stability, as long as oral health and regular oral hygiene is maintained. Secondarily, we tested the hypothesis that the supragingival microbiota in oral health is resistant against the external perturbation of regular use of probiotics.

## 2. Results

### 2.1. Background and Clinical Data

Background data of the study population are detailed in Table 1. While a comparable age distribution was observed between the two groups, the test group comprised a significantly higher amount of male participants (*p* < 0.05). Table 2 details the clinical data at each time-point. As can be seen, plaque index and bleeding on probing was stable during the three months observation period for both groups. Furthermore, no significant differences between groups were registered during the trial.

### 2.2. Side Effects and Compliance

A limited number of the participants reported discomfort in accordance to consuming the lozenges but at a minor level and all completed the trial. The mean number of missed lozenges was 8.9 calculated from remaining lozenges at the end of the trial and corresponding to one missed lozenge every 19th day.

### 2.3. Sequencing Metadata

A total of 31 samples failed quality control. Thus, a total of 41,936 reads per sample were included in downstream analysis, including 4285 unique OTUs, which were identified from 15.5 million reads retrieved from a total of 369 samples. A total of 402 different bacterial species and 109 bacterial genera were identified corresponding to 62.3% and 96.5% coverage of the generated sequences, respectively. A mean Alpha diversity as determined by Shannon index of 4.5 was observed. Positive frequency and relative abundance of the probiotic strains is detailed in Supplementary Figure S1A,B.

### 2.4. Long Term Stability of the Supragingival Microbiota in Oral Health

**Figure 1:**
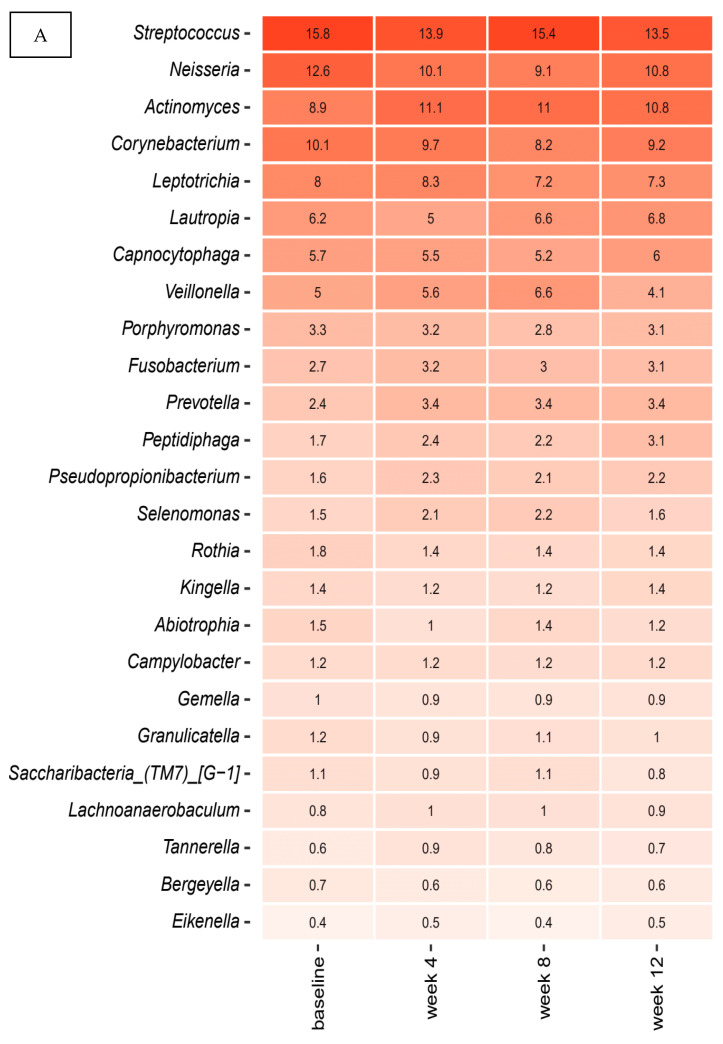

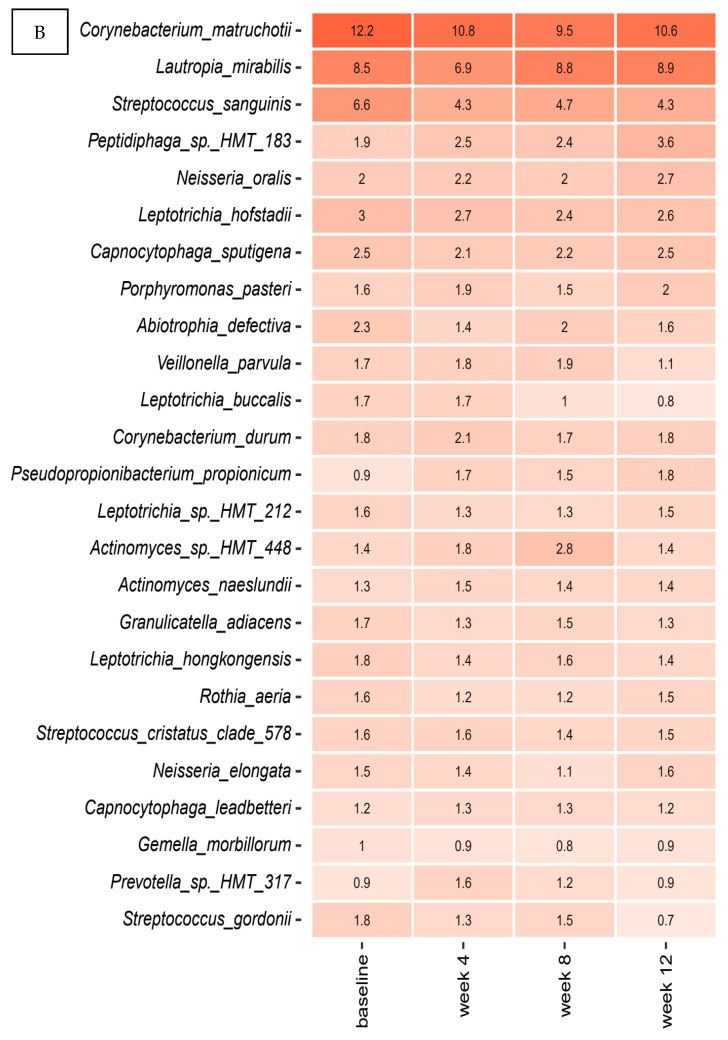

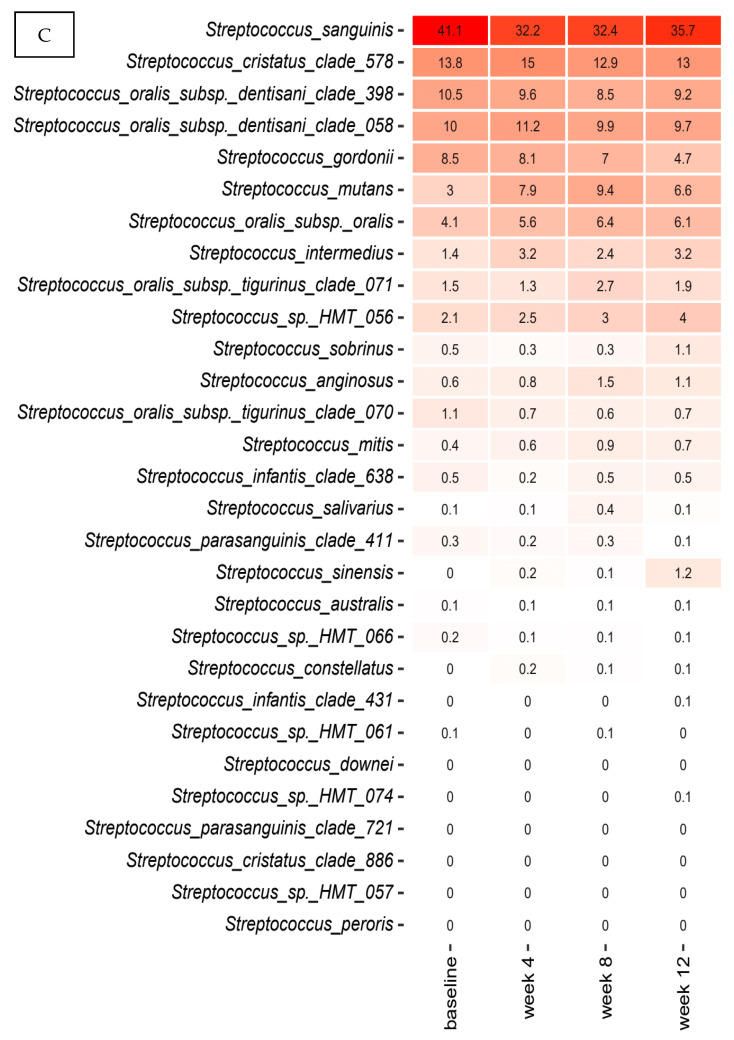
Predominant microbiota. Relative abundance expressed as mean values of top 25 predominant genera (**A**), species (**B**) and *Streptococcus* species (**C**) in the placebo group. The intensity of the red color denotes the level of relative abundance.

Figure 1A,B details relative abundance of the core supragingival microbiota at four different time points—baseline, week 4, week 8 and week 12—expressed by the mean value of predominant genera (Figure 1A) and species (Figure 1B) in the placebo group. As seen, the core supragingival microbiota, which constituted approximately 55% of the microbiota, was comprised of *Streptococcus*, *Neisseria*, *Actinomyces*, *Corynebacterium,* and *Leptotricia* species. The top 20 bacterial genera and species were highly stable, as no significant alterations in relative abundance were observed during the three months observation period.

Figure 1C details relative abundance of predominant *Streptococcus* species at each time-point in the placebo group. As can be seen, mean values of relative abundance of the most predominant *Streptococcus* species, *Streptococcus sanguinis*, *Streptococcus cristatus, Streotococcus oralis,* and *Streptococcus gordonii* were highly stable during the three months trial period.

**Figure 2:**
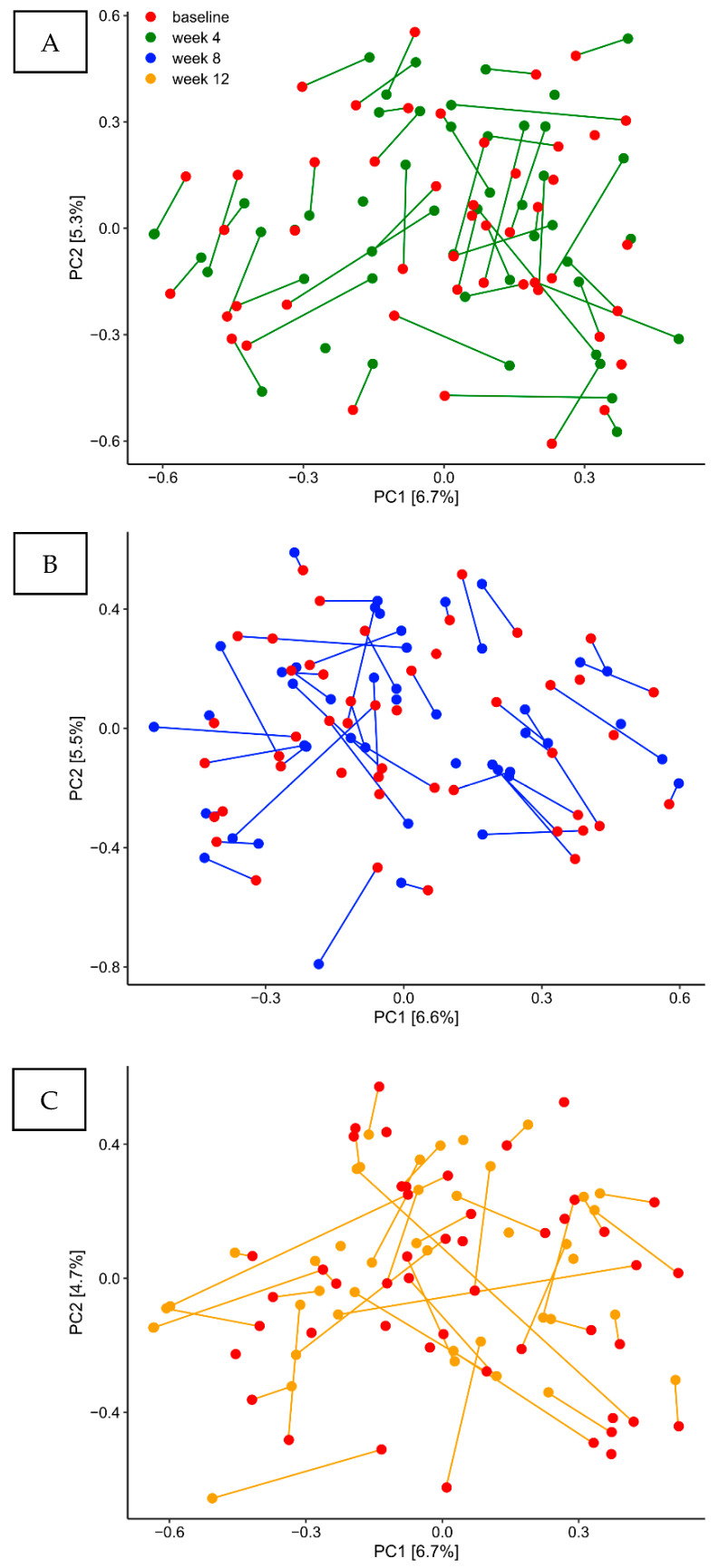
Predominant microbiota. Principal component analysis of the control group. PCA expressed by the two most decisive variables (PC1 and PC2) accounting for approx. 12% of the variation of the dataset in the placebo group. (**A**) baseline vs. week 4. (**B**) baseline vs. week 8. (**C**) baseline vs. week 12.

Samples from each time-point from the placebo group are visualized in Figure 2A–C by Principal Component Analysis (PCA) based on the two most decisive components (PC1 and PC2), which collectively covered approximately 12% of the variation of the dataset. PCA analysis clearly demonstrated completely random distribution of samples, which indicate that the core supragingival microbiota in the placebo group remained stable during three months observation period.

### 2.5. Probiotics Lozenges Had No Impact on the Composition of the Supragingival Microbiota

**Figure 3:**
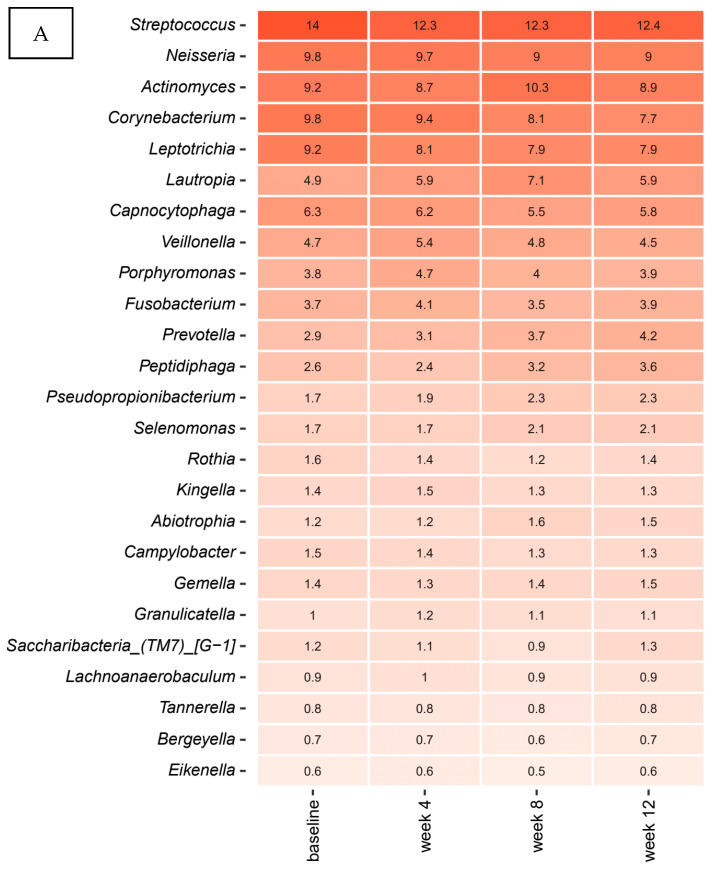

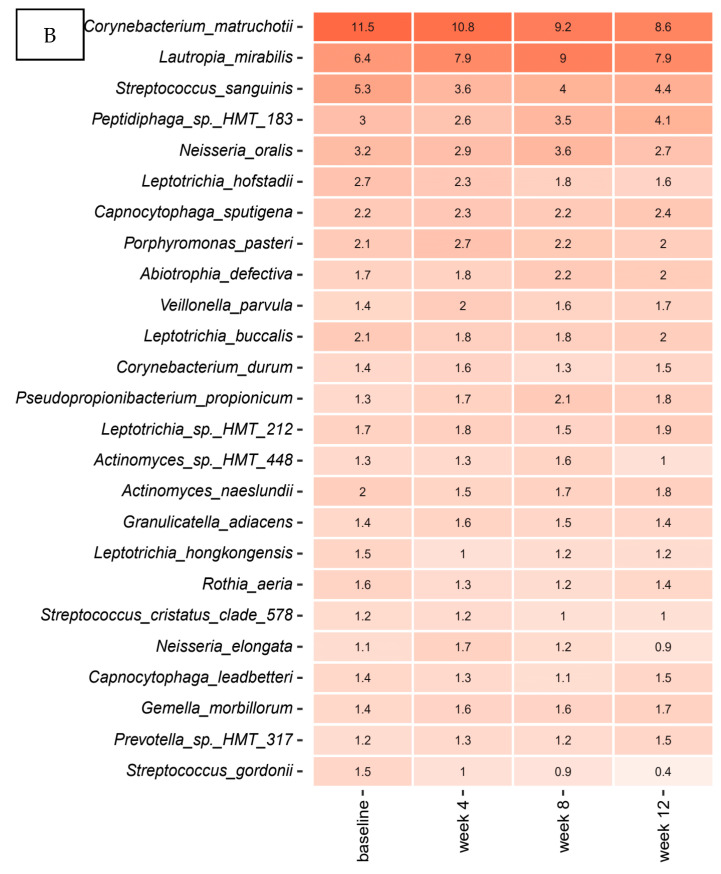

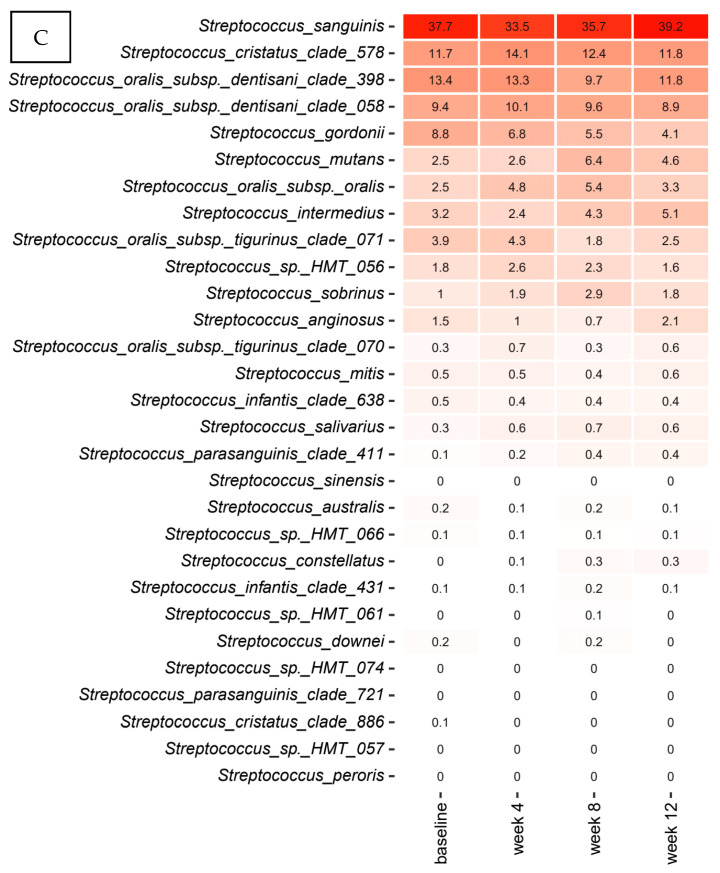
Predominant microbiota. Relative abundance expressed as mean values of top 25 predominant genera (**A**), species (**B**) and *Streptococcus* species (**C**) in the probiotics group. The intensity of the red color denotes the level of relative abundance.

Figure 3A,B details relative abundance of the core supragingival microbiota in the probiotics group recorded at the four different time-points (baseline, week 4, week 8, week 12), expressed as mean values of predominant genera (Figure 3A) and species (Figure 3B). The core supragingival microbiota in the probiotics group was also comprised of *Streptococcus*, *Neisseria*, *Actinomyces*, *Corynebacterium,* and *Leptotricia* species. The top 20 bacterial genera and species were highly stable in the probiotics group, as no significant alterations in relative abundance were observed during the three months intervention period. Further, no differences in the core microbiota, as determined by relative abundance of predominant bacterial genera and species, were observed between the probiotics group and the control group.

Figure 3C details relative abundance of predominant *Streptococcus* species at each time point in the probiotics group. As can be seen, mean values of relative abundance of the most predominant *Streptococcus* species, *S. sanguinis*, *S. cristatus*, *S. oralis,* and *S. gordonii,* was not influenced by use of probiotics, since relative abundance of the predominant *Streptococcus* species remained highly stable during the three months intervention period. Moreover, no differences in the predominant *Streptococcus* species, as determined by relative abundance, were observed between the probiotics group and the control group.

**Figure 4:**
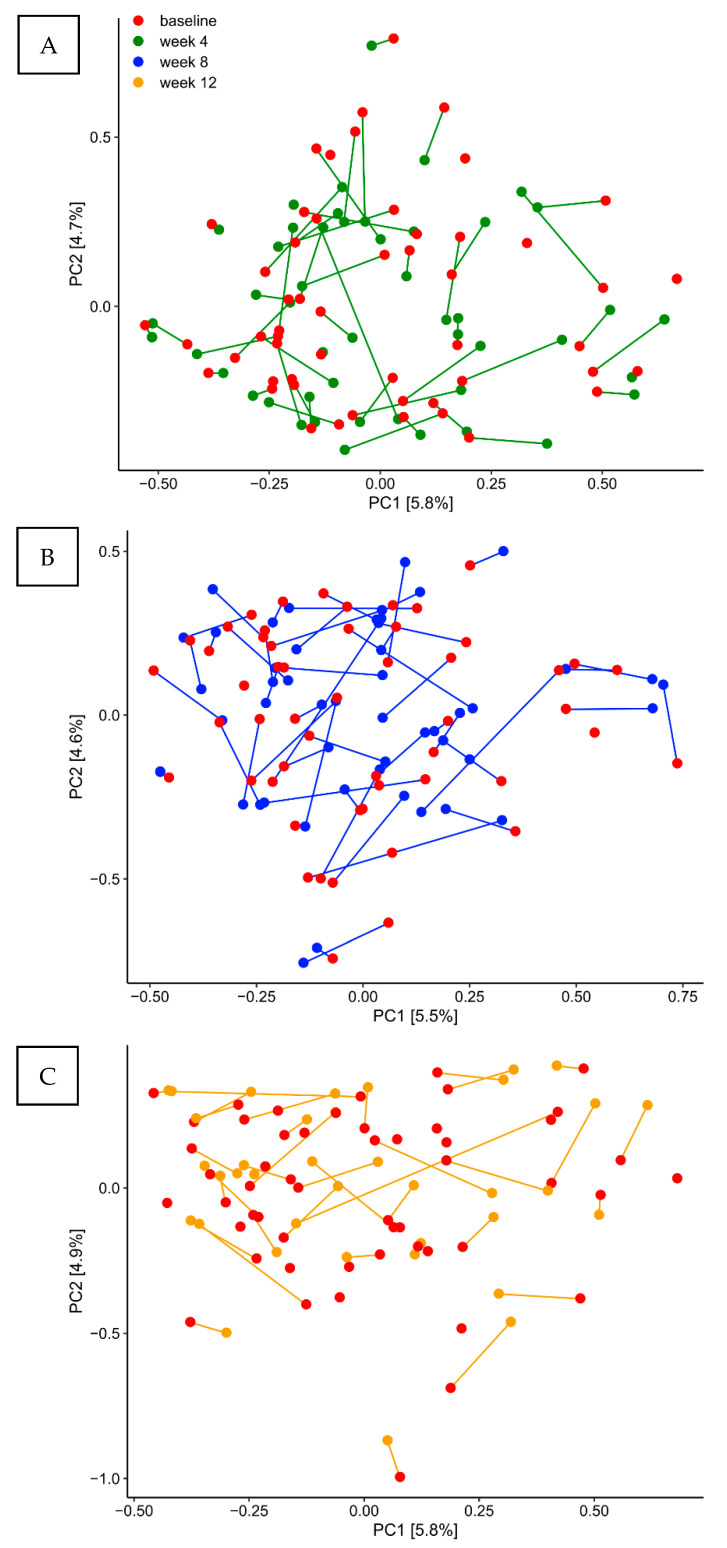
Principal component analysis of the probiotics group: PCA expressed by the two most decisive variables (PC1 and PC2) accounting for approx. 12% of the variation of the dataset in the probiotics group. (**A**) baseline vs. week 4. (**B**) baseline vs. week 8. (**C**) baseline vs. week 12.

PCA showed completely random distribution of samples from the probiotics group at each time-point, demonstrating that the core supragingival microbiota was not influenced by use of probiotics (Figure 4A–C). On the other hand, linear discriminant analysis Effect Size (LEfSe) analysis revealed that use of probiotics was associated with a significant increase in *Leptotrichia buccalis*, *Gemella morbillorum* and *Leptotrichia* species HMT 212 (Supplemental Figure S2).

## 3. Discussion

The purpose of the present study was to longitudinally characterize the supragingival microbiota throughout a three months period in orally healthy individuals. We tested the hypothesis that the supragingival microbiota shows a high degree of compositional stability, which is resistant against regular use of probiotics, as long as oral health is maintained. To the best of our knowledge, this is the first large scale clinical trial to characterize the supragingival microbiota in oral health.

The main finding was that the supragingival microbiota in orally healthy individuals shows a remarkable degree of compositional stability. Accordingly, no changes of the core microbiota, as expressed by relative abundance of predominant genera and species were evident during the three months observation period (Figure 1A,B). Moreover, principal component analysis revealed no tendency of sample clustering at different time points (Figure 2A–C). Notably, rampant alterations of the supragingival microbiota have been demonstrated alongside clinical changes, when oral hygiene is discontinued for a short period [10,31,32]. Likewise, compositional changes of the supragingival microbiota are critically involved in the pathogenesis of dental caries, where an increase in the most acidogenic members of the *Streptococcus* genus on expense of health related *Streptococcus* species is evident in the cariogenic biofilm [33]. In the present study we did not observe any longitudinal alterations of *Streptococcus* species (Figure 1C). Indeed, clinical recordings demonstrated very low levels of dental plaque and bleeding on probing, which remained stable throughout the study period (Table 2). Therefore, our data clearly demonstrate long term stability of the supragingival microbiota as long as oral health is maintained, which is in line with a previous small scale study that showed 96.6% comparability of consecutive supragingival plaque samples collected from 10 healthy individuals during a three months period [34]. Consequently, based on the long term compositional stability in oral health, the supragingival microbiota seems to be a relevant in-vivo model system to study interactions between polymicrobial biofilms and the host during coordinated perturbations.

In the present study, we did not identify any impact on the supragingival microbiota based on daily use of probiotics, i.e., *Lacticaseibacillus rhamnosus* (formerly *Lactobacillus*) PB01 DSM14870 and *Latilactobacillus curvatus* (formerly *Lactobacillus*) EB10 DSM32307. Accordingly, the selected probiotic strains did not induce any changes in relative abundance of predominant bacterial genera and species (Figure 3A,B). On the other hand, LEfSe analysis showed a significant increase in specific oral species, *Leptotrichia buccalis*, *Gemella morbillorum,* and *Leptotrichia* species HMT 212 after three months use of probiotics (Supplemental Figure S2). Notably, despite being anaerobic Gram-negative rods belonging to the phylum of Fusobacterium, *Leptotrichia* species have been described having proficient carbohydrate metabolism [35]. Accordingly, the selected probiotic strains are also capable of carbohydrate metabolism [36]. Therefore, the blossoming of *Leptotrichia* species suggests that use of probiotics might favor conditions of bacterial species with the same ecological preferences. However, this had no influence on the composition of predominant *Streptococcus* species (Figure 3C), which are the prime act in carbohydrate metabolism in the oral cavity. Therefore, data suggest that the dynamic metabolic resistance of the biofilm [37], was sufficient to counteract any ecological effect of the probiotic strains tested.

Several systematic reviews and meta-analyses have concluded that there seems to be a potential clinical effect for the adjunctive use of probiotics in treatment of dental caries and periodontitis [18,19,20,23,26]. However, in contrast to the majority of the studies included in the meta-analyses, our study population was comprised of orally healthy individuals with very low levels of dental plaque and gingival inflammation, which is why the clinical conditions were probably too stable for the probiotics to have a clinically recordable effect. Our data therefore suggest that the tested probiotics have no augmenting effect on the composition of the supragingival microbiota in orally healthy individuals with meticulous oral hygiene. On the other hand, it is possible that the tested probiotics could have an effect on the supragingival microbiota in clinical conditions with treatment-requiring oral disease, when the oral microbiota is stressed and dysbiotic conditions rule. However, this should be tested in future studies.

Only few studies have used 16S sequencing to investigate the effect of probiotics on the oral microbiota, and the results are conflicting. Accordingly, two earlier studies did not reveal any impact of the selected probiotic strains, which is in line with our data [28,30]. On the contrary, two other studies [27,29] observed a significant increase of health associated bacterial taxa in combination with a decrease in potential pathogens. Importantly, previous studies have tested different probiotic strains including *Lactobacillus, Bifidobacterium,* and *Streptococcus* species [18,19,20,23,26]. Indeed, each probiotic strain express different targeted effects, which include specificity for age, gender, diet, residence, and oral as well as general health status [38]. Beyond that, earlier studies have been performed in cohorts with different age and oral health status, using various administration forms, including pacifiers, tablets, lozenges, and milk. Obviously, the abovementioned methodological differences hamper the possibility to compare data between studies on the impact of probiotics on the oral microbiota. Therefore, there is a need for large scale clinical trials, which directly compare the effect of multiple probiotics strains on clinical, microbial and immunological parameters in different clinical conditions in the same population.

The present study has several limitations, including the use of pooled supragingival plaque samples, which limit the possibility to address any site specific impact of the probiotics tested. Indeed, anatomical characteristics have been described within the supragingival microbiota [39]. Therefore, to embrace site-specific variations we choose a robust sampling strategy, which included sampling from both buccal and lingual surfaces in both anterior and posterior regions of the oral cavity. Another important limitation was that the molecular method used, did not offer resolution more specific than the species level. Notably, considerable fluctuations of *Streptococcus* species have previously been revealed at the clonal level [40]. However, as the method used in the present did not offer clonal level resolution, we were not able to evaluate stability of the supragingival microbiota at clonal level. The main strength of the present study is the randomized study design and the large study population, which to the best of our knowledge is the largest used for studies on the effect of probiotics on the oral microbiota. On the other hand, our study population turned out to be extremely homogenous, which obviously limits the generalizability of the data presented. Finally, as a consequence of a recently published paper [41], which demonstrated an impact of toothpaste on the composition of the supragingival microbiota, we tried to control any impact from toothpaste. Therefore, we included a four-week wash-out period in the study design, where all participants were handed the same toothpaste (Zendium Classic^TM^) and instructed to continue usage throughout the trial period. It is therefore possible that any potential influence from the probiotics tested might have been hidden by the effect of the toothpaste used by the participants.

In conclusion, data from the present study clearly demonstrates long term compositional stability of the supragingival microbiota as long as oral health is maintained. Therefore, data supports the use of the supragingival microbiota as a model system to study the impact of internal and external perturbations on polymicrobial biofilms. In addition, the tested probiotics had no augmenting effect on the supragingival microbiota in oral health. Further studies are needed to evaluate the potential of using these probiotics strains in clinical conditions, where the supragingival microbiota is stressed by perturbations, such as oral hygiene discontinuation or excessive sugar consumption.

## 4. Materials and Methods

### 4.1. Study Design

**Figure 5:**
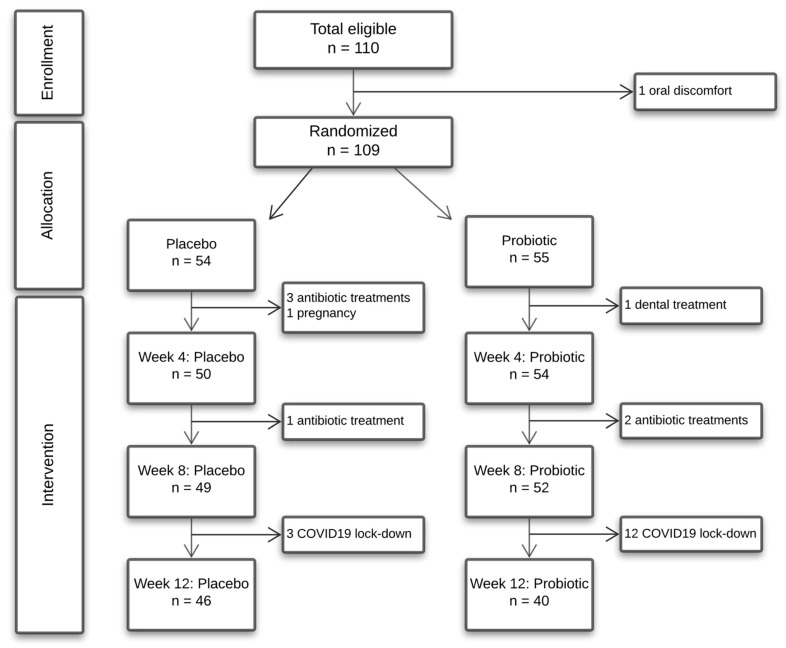
Flowchart of the study.

The present study was a double-blinded, randomized, placebo-controlled, clinical trial with a total duration of 16 weeks, performed from November 2019 to March 2020. Before randomization, all participants completed a 4-week wash-out period, where they all used the same toothpaste (Zendium Classic™), which was subsequently used throughout the study period. At baseline, computerized randomization (www.randomizer.org, accessed on 1 October 2019) was performed to allocate participants to either receive probiotic (test group) or placebo lozenges twice a day for the subsequent 12 weeks of intervention. Clinical examination and sample collection were performed at baseline, and after 4, 8, and 12 weeks, respectively. Timeline of the study is detailed in Figure 5.

### 4.2. Study Population

The study population comprised a total of 110 oral and systemic healthy individuals aged 19–31 years, distributed in a probiotic (*n* = 55) and placebo (*n* = 55) group. The sample size was based on results from a previous paper [41]. In order to disclose significant changes in the oral microbiota, a power calculation with α = 0.05 and β = 0.2 revealed that 50 subjects was needed in each group. To compensate for dropouts, we enrolled a total of 110 subjects in this project. General exclusion criteria were; treatment requiring dental caries and/or periodontitis, extensive gingivitis, current smoking, pregnancy, systemic diseases, and use of any systemic antibiotics three months prior to study participation. Participants were recruited at Department of Odontology, University of Copenhagen.

A total number of 24 participants did not complete the trial, with the main part being due to the COVID-19 lockdown at week 12. The distribution of dropouts was as following: baseline (*n* = 1), week 4 (*n* = 3), week 8 (*n* = 5), and week 12 (*n* = 15). The reasons for dropout were as follows: COVID-19 lockdown (*n* = 15), antibiotic prescription (*n* = 6), pregnancy (*n* = 1), surgical removal of tooth (*n* = 1), extensive mucosal irritation without relation to lozenges (*n* = 1) (Figure 5). All participants signed informed consent prior to participation, and the study was performed in accordance with the Helsinki declaration. The study was approved by the regional ethical committee (H-19086532) and reported to the local data authorization of the Faculty of Health and Medical Sciences, University of Copenhagen (514-0434/19-3000). Finally, the study was registered at ClinicalTrials.gov (UCPH_OI_003).

### 4.3. Clinical Examination

Clinical oral examination was performed by the same examiner (C.L.-O.) at baseline and after 4, 8, and 12 weeks, respectively. Calibration between C.L.-O. and D.B. was performed at baseline. Full mouth clinical examination included systematic registration of dental plaque formation and bleeding on probing at six sites per tooth (third molars excluded). Plaque levels were registered using TePe PlaqSearch™ (Malmo, Sweden) disclosing tablets at three equal areas at each tooth surface by use of the Modified Quigley and Hein index [42], which is graduated from 0 to 5 (0: no plaque, 1: speckles of plaque along the gingival margin, 2: a continuous line of plaque up to 2 mm in depth along the gingival margin, 3: plaque covered up to 1/3 of the assessment area, 4: plaque covered up to 2/3 of the assessment area, 5: plaque covered the entire assessment area). Gingival bleeding was likewise registered at six sites per tooth and graduated from 0 to 2 (0: no bleeding, 1: bleeding within 30 s of probing, 2: spontaneous bleeding) as previously described [43]. Subsequently, a plaque index (PI) and a gingival bleeding index (BI) were calculated.

### 4.4. Collection of Samples

Originally, 440 supragingival plaque samples should have been collected throughout the trial period, but due to periodic drop-outs and especially COVID-19 a total of 400 supragingival plaque samples were collected at baseline (*n* = 109), week 4 (*n* = 104), week 8 (*n* = 101) and week 12 (*n* = 86), respectively. Participants were instructed to refrain from oral hygiene procedures at the day of sampling. In general, samples were collected between 8.00 a.m. and 17.00 p.m. Monday–Friday, but great effort was made to accommodate that each participant was sampled approximately at the same point of time at each visit. The supragingival plaque samples were collected from the lingual surface of molars and incisors in the lower jaw, and the buccal surface of molars and canines in the upper jaw as previously described [10], and subsequently pooled and suspended in 1 mL saline. All samples were as soon as possible stored at −80 °C until further analyses.

### 4.5. Probiotics and Placebo

The probiotic and placebo tablets were the same as used in our recent study [28] and contained an equal mix of *Lacticaseibacillus rhamnosus* (formerly *Lactobacillus*) PB01 DSM14870 and *Latilactobacillus curvatus* (formerly *Lactobacillus*) EB10 DSM32307 with a concentration of 1·10^9^ CFU/tablet. The strains where chosen based on both in vitro assays that investigated the immunologic and growth-inhibiting effect on proposed periopathogens (data not shown), and the above-mentioned in vivo study. The probiotic and placebo tablets were identical in size and composition but without the addition of the probiotic strains to the placebo tablets. The tablets were packed and handed out to the participants in identical pots only marked with participant number, whose distribution in the probiotic- and placebo group remained blinded to both participants as well as examiner throughout the trial period. Both tablets were manufactured and provided by Deerland Probiotics and Enzymes A/S, Hundested, Denmark. Participants were instructed to soak and distribute one tablet in the oral cavity twice a day, morning and evening, immediately after oral hygiene procedure to ensure maximum concentration of the probiotic strains during the initial biofilm formation. Furthermore, participants were instructed to avoid any food or drinking consumption the subsequent 30 min to minimize the influence of oral clearance and achieve the longest and thus best possible effect of the probiotic tested.

### 4.6. DNA Extraction

DNA was purified from untreated saliva and plaque samples using the ZymoBIOMICS 96 DNA-kit, as instructed by the manufacturer (Cat.#D4309, Zymo Research, Irvine, CA, USA). Briefly, samples were thawed and diluted 1:4 in DNA/RNA Shield (Cat.#R1100-250, Zymo Research, Irvine, CA, USA) in the included ZR BashingBead Lysis Tubes. The ZR BashingBead Lysis Tubes were subsequently vortexed at full speed for 40 min in a Horizontal-(24) Microtube Holder mounted on a Vortex Genie 2 (Scientific Industries, Bohemia, NY, USA). After lysis, all samples were frozen at −20 °C until DNA extraction following the manufacturers protocol with optional purification using the included Silicon-A-HRC plate.

### 4.7. Library Preparation

Bacterial 16S V1-V3 rRNA gene sequencing libraries were prepared by a custom protocol based on (Caporaso et al., 2012) [44]. Up to 10 ng of extracted DNA was used as template for PCR amplification of the bacterial 16S V1-V3 rRNA gene amplicons. Each PCR reaction (25 μL) contained (12.5 μL) PCRBIO Ultra mix (PCR Biosystems, Wayne, PA, USA) and 400 nM of each forward and reverse tailed primer mix. PCR was conducted with the following program: Initial denaturation at 95 °C for 2 min, 30 cycles of amplification (95 °C for 15 s, 55 °C for 15 s, 72 °C for 50 s) and a final elongation at 72 °C for 5 min. Duplicate PCR reactions were performed for each sample and the duplicates were pooled aft er PCR. The adaptors contain 16S V1-V3 specific primers: [27F] AGAGTTTGATCCTGGCTCAG and [534R] ATTACCGCGGCTGCTGG [45]. The resulting amplicon libraries were purified using the standard protocol for Agencourt Ampure XP Beads (Beckman Coulter, Brea, CA, USA) with a bead to sample ratio of 4:5. DNA was eluted in 25 μL of nuclease free water (Qiagen, Hilden, Germany). DNA concentration was measured using Qubit dsDNA HS Assay kit (Thermo Fisher Scientific, Waltham, MA, USA). Gel electrophoresis using TapeStation 2200 and D1000/High sensitivity D1000 screen tapes (Agilent technologies, Santa Clara, CA, USA) was used to validate product size and purity of a subset of sequencing libraries.

### 4.8. DNA Sequencing

The purified sequencing libraries were pooled in equimolar concentrations and diluted to 2 nM. The samples were paired-end sequenced (2 × 300 bp) on a MiSeq (Illumina, San Diego, CA, USA) using a MiSeq Reagent kit v3 (Illumina, San Diego, CA, USA) following the standard guidelines for preparing and loading samples on the MiSeq and sequencing approximately 100,000 reads/sample. >10% PhiX control library was spiked in to overcome low complexity issues often observed with amplicon samples.

### 4.9. Bioinformatic Processing

The base called and demultiplexed Illumina reads were processed using a usearch11 pipeline [46] using forward reads only matching against the 16S rRNA Human Oral Microbiome RefSeq database (HOMD) v. 15.2 [47]. The entire workflow can be summarized in the following steps, all with default settings unless otherwise noted: (1) PhiX spike in sequences were first filtered from each sample using the usearch11 filter_phix command. (2) The sequences were then filtered based on Q scores using [48] the usearch11 fastq_filter command with max expected errors set to 1.0 (fastq_maxee 1.0) and truncated to 250 bp (fastq_trunclen), and afterwards concatenated into a single fastq file. (3) The file with all quality checked reads were then dereplicated by using the usearch11 fastx_uniques command and afterwards zero radius operational taxonomic units (zOTUs), aka amplicon sequence variants (ASVs), were generated using the unoise3 command. (4) Taxonomy of the ASVs were then predicted using the SINTAX algorithm [49] (usearch11 sintax) with the settings strand both and sintax_cutoff 0.8 using the HOMD database. (5) Finally, an abundance table was generated using the usearch11 otutab command by mapping the zOTUs obtained from step 3 to the PhiX filtered reads from step 1. The results were analyzed in R v. 4.0.2 [50] through the Rstudio IDE using the ampvis package v.2.6.6. LEfSe (Linear discriminant analysis EffectSize) [51] was used to determine features (organisms) most likely to explain differences between all combinations of classes (interventions and timepoints). The analysis was carried out with default settings.

### 4.10. Statistics

Background data of the groups were compared using *t*-test and Fisher’s exact test. Clinical data, plaque index and bleeding index, were compared within groups using paired ANOVA and between groups using *t*-test. For these analyses, a *p*-value < 0.05 was considered significant. The microbiota of the supragingival plaque was characterized and compared by relative abundance, bacterial diversity, and visualizing of data by principal component analysis (PCA). Data on relative abundance were corrected for multiple dependent associations using Benjamini–Hochbergs correction [52].

## Figures and Tables

**Table 1:** Background data of the study group.

	Probiotics (*n* = 55)	Placebo (*n* = 55)
Gender female/male	37/18	49/6
Age (mean, range) years	23.4 (19–29)	24.1 (19–31)
Dental professions *	49/55	52/55

* Dental students, dental hygienist students, dentists, dental hygienists, dentist’s assistants.

**Table 2:** Clinical data of the study groups expressed as mean and range.

	Mean Plaque Index	Mean BI Index	BOP%
Baseline:	1.84 (0.35–2.97)	0.04 (0.00–0.17)	3.74 (0.00–16.67)
Probiotics (*n* = 55):	1.89 (0.68–2.97)	0.04 (0.00–0.17)	3.95 (0.00–16.67)
Placebo (*n* = 54):	1.79 (0.35–2.93)	0.04 (0.00–0.11)	3.55 (0.00–10.71)
Week 4:	1.79 (0.93–3.18)	0.07 (0.00–0.25)	6.27 (0.00–25.00)
Probiotics (*n* = 54):	1.81 (0.93–3.18)	0.07 (0.01–0.23)	7.10 (0.60–23.21)
Placebo (*n* = 50):	1.77 (0.99–2.43)	0.06 (0.00–0.25)	6.01 (0.00–25.00)
Week 8:	1.82 (0.65–2.77)	0.09 (0.00–0.27)	8.73 (0.00–27.38)
Probiotics (*n* = 52):	1.83 (0.97–2.77)	0.09 (0.02–0.23)	9.10 (1.79–23.21)
Placebo (*n* = 49):	1.81 (0.65–2.46)	0.10 (0.00–0.27)	9.75 (0.00–27.38)
Week 12:	1.69 (0.77–2.48)	0.06 (0.00–0.19)	4.49 (0.00–19.01)
Probiotics (*n* = 40):	1.71 (0.88–2.48)	0.06 (0.01–0.19)	5.91 (0.60–18.45)
Placebo (*n* = 46):	1.68 (0.70–2.35)	0.05 (0.00–0.19)	5.42 (0.00–19.05)

## Data Availability

Raw sequences have been deposited in European Nucleotide Archive (ENA, www.ebi.ac.uk) with the accession number PRJEB43048.

## Supplementary Materials

The following are available online at https://www.mdpi.com/2076-0817/10/4/391/s1, Figure S1: Identification of probiotic strains, Figure S2: Probiotics-associated bacterial species.
