# Supplementary material for: Probiotics Do Not Alter the Long-Term Stability of the Supragingival Microbiota in Healthy Subjects: A Randomized Controlled Trial
Source: Pathogens. 2021 Mar 24;10(4):391. doi: 10.3390/pathogens10040391 (PMC8064340; doi:10.3390/pathogens10040391)
Supplement: Supplementary file 1 [file pathogens-10-00391-s001.pdf]

A

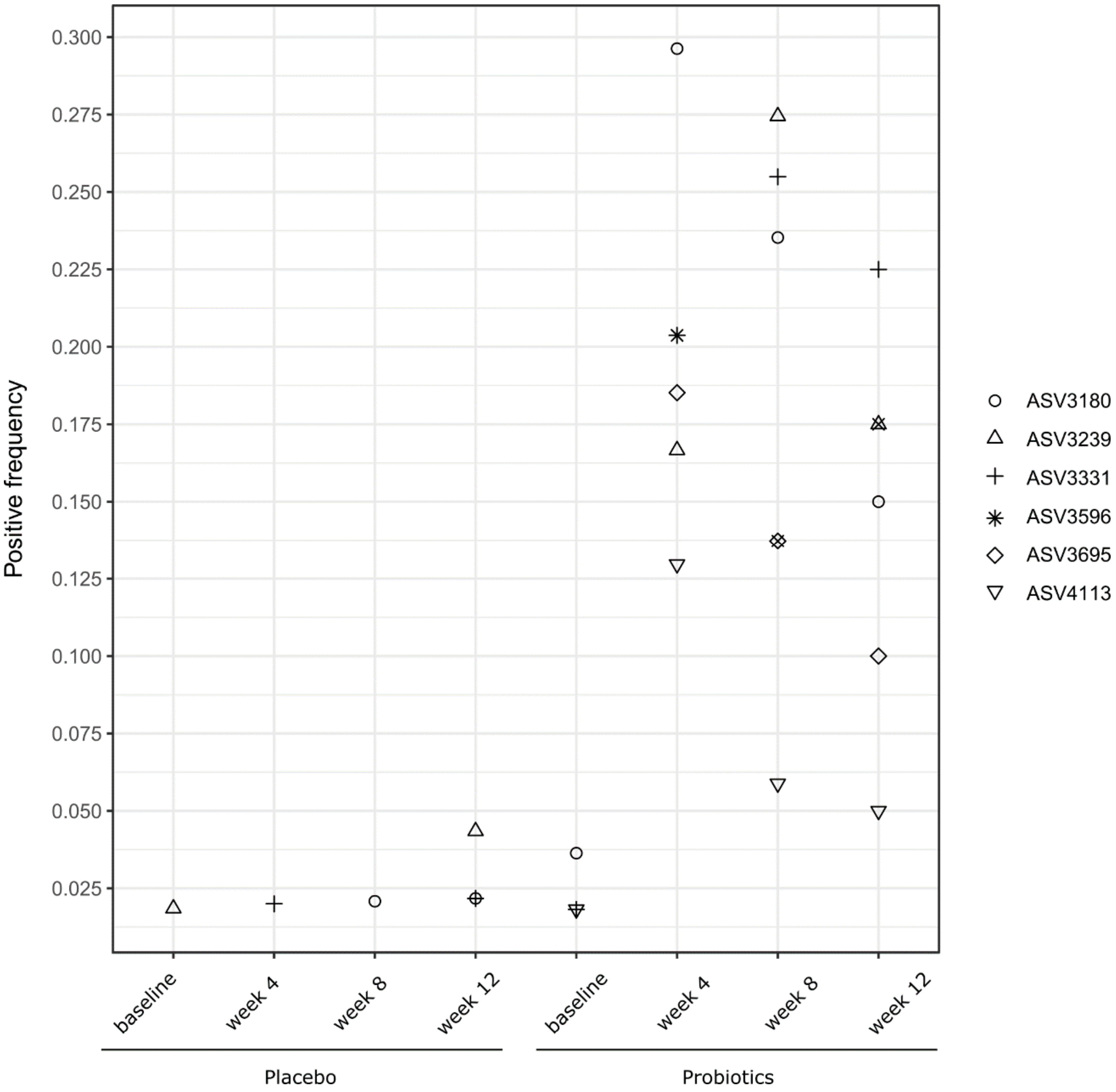

Positive frequency
0.300
0.275
0.250
0.225
0.200
0.175
0.150
0.125
0.100
0.075
0.050
0.025
baseline
week 4
week 8
week 12
baseline
week 4
week 8
week 12
Placebo
Probiotics
ASV3180
ASV3239
ASV3331
ASV3596
ASV3695
ASV4113

B

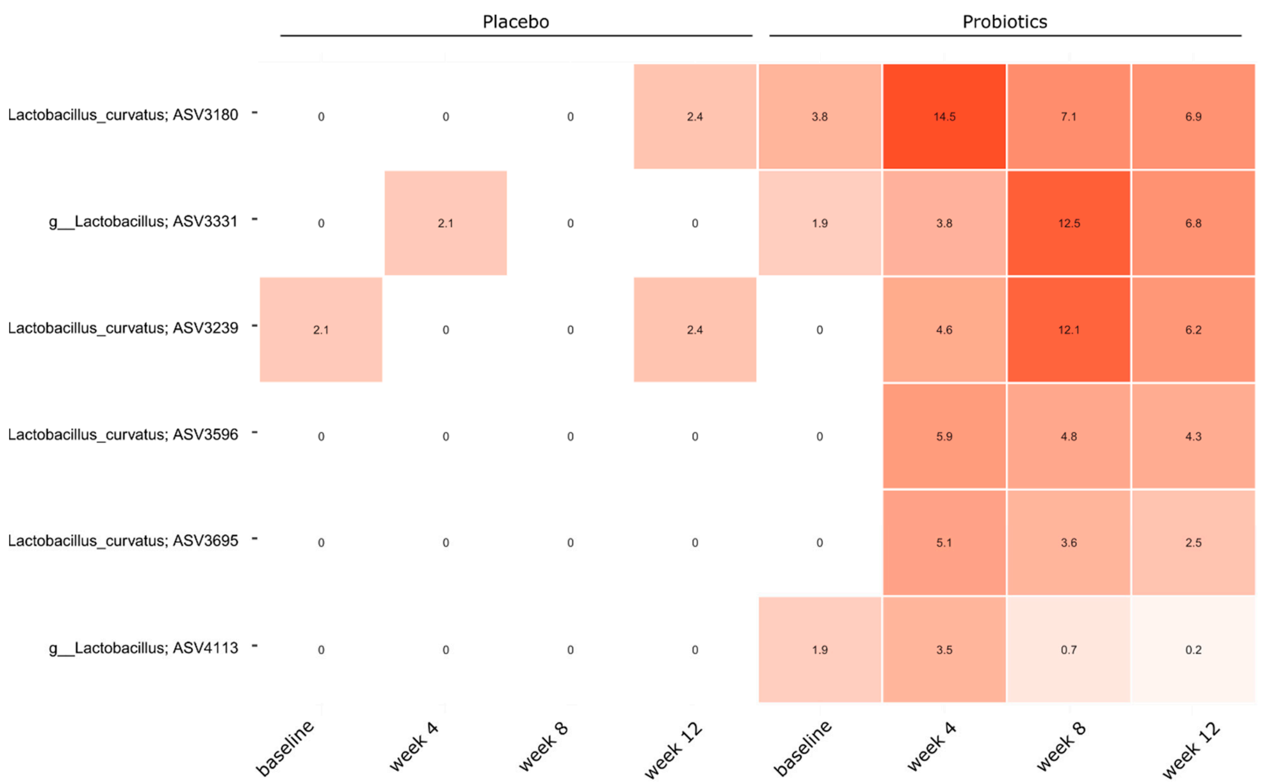

Figure S1. Identification of probiotic strains. Probiotic strains were identified using six reference ASVs (ASV3180, ASV3239, ASV3331, ASV3596, ASV3695 and ASV4113). (**A**) Positive frequency of each ASV in the placebo and the probiotics group at baseline, week 4, week 8 and week 12. (**B**) Relative abundance of the six ASVs expressed as percentage of total abundance of genus *Lactobacillus* in the placebo and the probiotics group at baseline, week 4, week 8 and week 12.

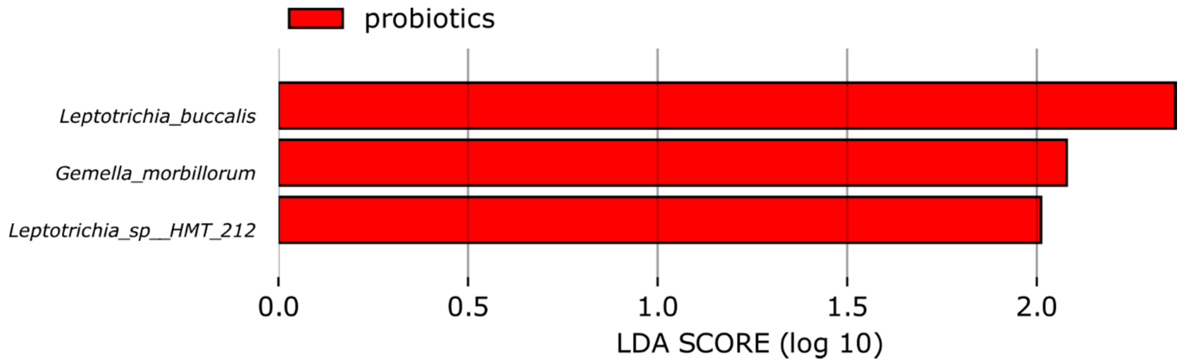

Figure S2. Probiotics-associated bacterial species. Linear discriminant analysis Effect Size (LEfSe) analysis demonstrating bacterial species associated with samples collected from the probiotics group versus the placebo group at week 12.
